# A machine learning framework for estimating the probability of blacklegged tick population establishment in eastern Canada using Earth observation data

**DOI:** 10.1371/journal.pone.0332582

**Published:** 2025-09-22

**Authors:** Hamid Ghanbari, Kevin Siebels, Ariane Dumas, Emily S. Acheson, Catherine Bouchard, Kirsten Crandall, Patrick A. Leighton, Nicholas H. Ogden, Erin E. Rees

**Affiliations:** 1 Modelling Hub Division, Applied Public Health Sciences Directorate, Science and Policy Integration Branch, Public Health Agency of Canada, Quebec, Canada; 2 Groupe de Recherche en Épidémiologie des Zoonoses et Santé Publique, Université de Montréal, Quebec, Canada; 3 Département de Pathologie et microbiologie, Faculté de médecine vétérinaire, Quebec; 4 Unité Maladies Infectieuses en communauté, Direction des risques biologiques, Institut national de santé publique du Québec, Quebec; University of North Dakota School of Medicine and Health Sciences, UNITED STATES OF AMERICA

## Abstract

*Ixodes scapularis* ticks are the primary vector of Lyme disease (LD) in North America, and their range has expanded into southeastern and southcentral Canada with climate change. This study presents a comprehensive machine learning (ML) framework to estimate the probability of blacklegged tick population establishment as measured using active tick surveillance data. Environmental predictor variables were derived from Earth observation (EO) data at multiple spatial scales to assess their individual contributions in the prediction models. Among the tested ML algorithms, XGBoost emerged as the top-performing model, achieving high sensitivity (0.83) and specificity (0.71) in predicting population establishment. Performance was optimized when using predictor variables derived from a 1 km radius around surveillance sites. Top predictors included cumulative annual degree-days above 0°C and maximum temperature of warmest month, reflecting the importance of temperature in enabling tick survival and reproduction. Additional predictor variables of high importance included silty soil (lower clay content) with slightly higher than average SOC and pH, and land cover types that contained broadleaf forests (percent mixed forest, percent broadleaf) and less urban areas. By integrating ML with open access EO data, this study demonstrates that accurate, easily updatable risk maps can be produced to support public health management of LD, and more broadly, the growing threat of tick-borne diseases in a changing climate.

## Introduction

The blacklegged tick, *Ixodes scapularis* (Say), is a vector of several pathogens of public health concern. It transmits *Borrelia burgdorferi* sensu stricto (termed *B. burgdorferi* in this article), the bacterium responsible for Lyme disease (LD). In North America, LD is the most common vector-borne disease in humans [1,2]. Blacklegged ticks are widely distributed in the central and eastern United States and have expanded their range northwards into central and eastern Canada. The range expansion is largely driven by climate change, which has created more favourable conditions for tick population survival [3,4]. In this context, migratory birds import ticks from source locations and the population can become established [5]. There is typically a lag of approximately five years between the first signs of population establishment and the onset of efficient *B. burgdorferi* transmission cycles [6,7]. Infection prevalence increases over several years, potentially reaching up to 50% [8]. This combination of increased geographic range and increasing infection prevalence has resulted in an almost exponential increase in human LD cases since 2009 [9].

Using a One Health approach, Canada has utilized opportunistic “passive” surveillance data (i.e., ticks submitted by the public), active tick surveillance data, and human case data to identify emerging risk areas for LD (e.g., [10–12]). Accurate and fine-scale identification of areas where tick populations are emerging (before there is significant risk of LD) and established (with significant LD risk) supports public health management to develop timely and targeted preventative and response actions. Preventative measures include public health messaging to raise awareness of emerging risks and advance notice to healthcare providers in affected areas. Response actions include the use of accurate risk maps to help clinicians assess whether patients have visited high-risk areas, particularly when LD diagnosis is challenging due to inconclusive test results but typical symptoms are present. Active field surveillance is the gold standard method for determining the distribution of the tick population and its relative abundance [13], but it is highly resource intensive, and is therefore too limited in coverage to fully assess the presence of tick populations at a broad scale in Canada. Ecological niche modelling (ENM) can use active surveillance data to identify the environmental suitability for ticks in terms of climate and habitat conditions [14]. From ENMs, risk maps can be created that support public health efforts for LD and other *I. scapularis* transmitted diseases.

The *I. scapularis* species distribution is influenced by specific biotic (e.g., vegetation cover, forest types, suitable animal hosts) and abiotic (e.g., temperature, soil properties, and humidity) conditions, at both macro- and the micro-environmental scales [15]. Laboratory and field studies have elucidated the role of temperature as a key determinant of the occurrence and abundance of *I. scapularis*. Temperature influences the duration of the tick lifecycle by affecting the speed of interstadial development between life stages [3,16]. A convenient index that reflects the temperature requirements of the multi-year tick lifecycle can be derived from cumulative annual degree-days above 0°C (DD > 0°C). Below an approximate threshold of 2800 DD > 0°C, *I. scapularis* populations cannot establish or persist because the temperatures are too cold for the ticks to complete their lifecycle [17].

Even when the DD > 0°C temperature threshold is exceeded, successful population establishment also depends on additional factors. Ticks are susceptible to desiccation, especially in the early stages of their life cycle [18,19]. Humidity is essential for ticks to regulate water balance and survive during their prolonged off-host periods [19]. Ticks have developed behavioural and physiological adaptations such as seeking refuge in moist environments (e.g., forest leaf litter layer) or entering diapause [20–22]. Forested habitats are essential for tick survival during temperature extremes and drought [23]. Although leaf litter significantly contributes to overwinter survival, snow cover may be also be protective in colder regions by providing insulation [24–27].

Additional environmental predictor variables found to associate with tick populations include topographical features and soil attributes [28,29]. A study conducted in southern Quebec reported that small differences in elevation (35–215 m) were negatively associated with nymphal density. This association may reflect simple temperature effects [30] or other unmeasured factors along the elevation gradient [31]. In a study conducted in the United States, north-facing slopes had higher tick abundance possibly due to the lower evapotranspiration given less exposure to solar radiation [29]. Soil properties, such as soil texture, amount of organic matter, and pH may also affect the survival and behaviour of ticks directly and indirectly by providing refuge from extreme weather conditions and supporting host habitat [32–34]. Soil hydrology, in particular, plays a significant role in tick survival and distribution. In arid regions, soils with higher moisture retention, such as those rich in silt, are more favourable for ticks due to their ability to maintain humidity [35]. Conversely, in wetter environments prone to inundation, well-draining sandy soils can support tick populations by reducing excessive soil water content that can be lethal to ticks [36].

In this study, we applied an ENM approach to estimate the probability of blacklegged tick population establishment in Quebec and Ontario, Canada, using environmental predictors primarily obtained from Earth observation (EO) data. Satellite EO data and meteorological stations provide extensive and multi-scale information that can be assessed for linkages to tick ecology using biologically plausible predictors variables from climate, habitat, topography, and soil [14,37–39]. For public health applications, EO-derived model predictors are particularly useful when the data are open-access, frequently updated, and covering large geographic regions. This enables models to be readily maintained and applied elsewhere. Specifically, we assessed a range of statistical and ML models to assess how predictor variables, derived at multiple spatial scales, associated to a binary outcome of tick population establishment, as measured using active surveillance data. The models were trained, validated and evaluated using data from Quebec, with spatial transferability of the final predictive model evaluated in Ontario.

## Materials and methods

### Tick surveillance data

**Fig 1:**
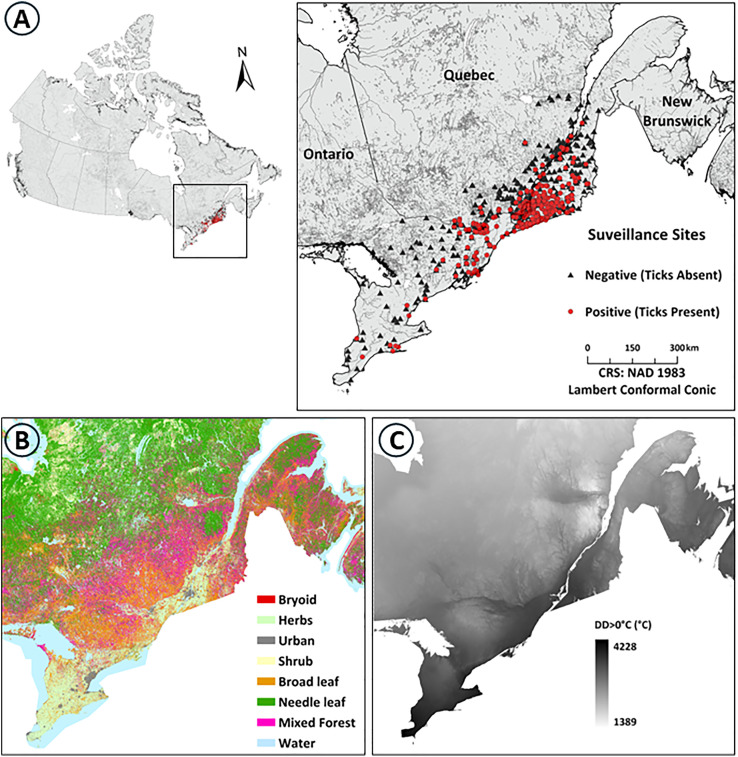
Geographic distribution of surveillance sites and environmental context. A) Distribution of tick surveillance sites across Quebec (2014-2023) and Ontario (2015-2018), B) land cover classification map, and C) DD > 0°C for 2022.

Active tick surveillance data from 933 sampling locations in Quebec, Canada between the years 2014 and 2023 were used to train, validate and evaluate the models (Fig 1A). The Quebec data were obtained from long-term surveillance database curated by the *Institut national de santé publique du Quebec* (INSPQ), where ticks were collected by the research team at *Université de Montréal*. A standard drag sampling method was implemented using a 1-m^2^ flannel cloth [40]. Sampling started with positioning four transects of 500 m using GPS coordinates. Tick dragging was then conducted along two sets of parallel transects: one set near the trail edge (1–5 m away) and the other set 25 m perpendicular into the forest. Each set of transects usually covered a 1 000 m distance out and back, totaling 2 000 m^2^ per site. Flannels were checked for ticks every 25 m along the transect. Collected ticks were then stored in 70% ethanol-filled tubes labeled by the collection site and sent to the Quebec Public Health Laboratory (*Laboratoire de santé publique du Quebec*) where ticks were identified by species. The recorded data at each site included the tick abundance (i.e., the number of counted ticks standardized by the dragging area) for three life stages (larvae, nymphs, and adults) accompanied by corresponding metadata including collection dates and GPS coordinates (centre location between transects’ start and end points).

**Fig 2:**
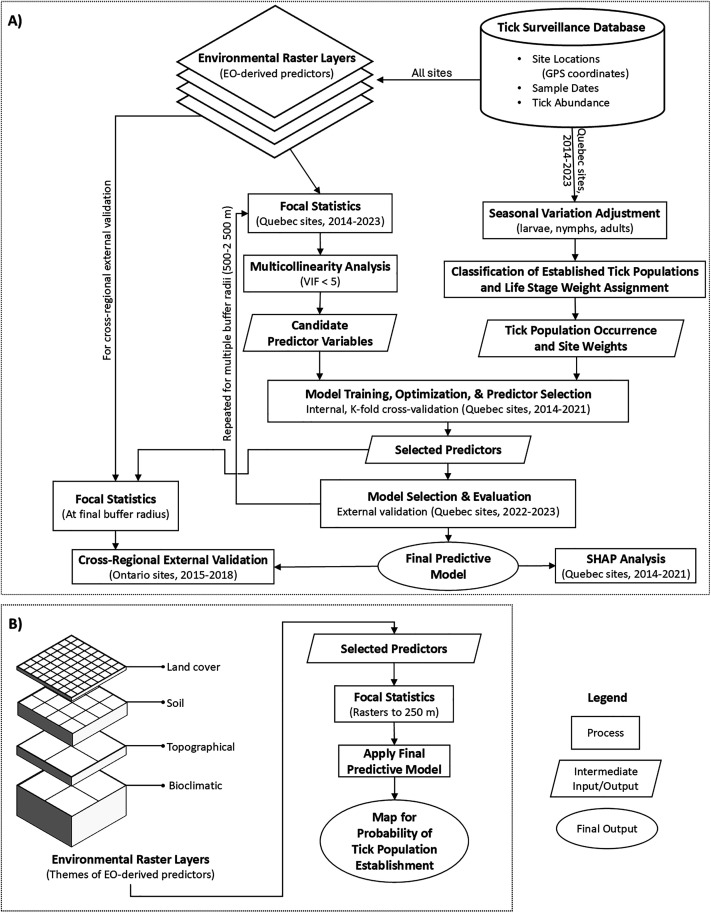
Workflow for estimating the probability of established tick populations. (A) Model development process; (B) Model implementation to produce a map showing the probability of tick population establishment at 250 m resolution.

In addition to the Quebec surveillance data, active surveillance data from 155 sampling locations in Ontario, during 2015–2018, were used for cross-regional external validation of the final predictive model (Figs 1A and 2). Data were collected following a similar and standard sampling protocol as used in Quebec [40,41].

### Candidate predictor variables

Initially, we considered 42 candidate predictor variables representing land cover types, biomass, bioclimate, topographical features, and soil attributes (Table 1). The data sources were selected based on the closest available data to the study scale, the time span and resolution of the surveillance data, and the applicability to other regions for future analyses.

**Table 1:** Candidate predictor variables included in the initial parameter selection.

Source (spatial resolution)	Variables (unit)	Attribute links
SCANFI (30 m)	percent bryoid (%)	Sums to 100%
	percent herbs (%)	
	percent urban (%)	
	percent shrub (%)	
	percent broadleaf forest (%)	
	percent needleleaf forest (%)	
	percent mixed forest (%)	
	percent water (%)	
	aboveground biomass (tons/ha)	None
	canopy height (m)	
		crown closure (%)
SRTM (30 m)	elevation (m)	None
	slope (degree)	
	aspect (degree)	
SoilGrids (250 m)	clay (%)	Sums to 100%
	sand (%)	
	silt (%)	
	BD (bulk density; kg/m^3^)	None
	pH	
		SOC (soil organic carbon; %)
Daymet (1 km)	bio1 (annual mean temperature; °C)	None
	bio2 (mean diurnal range; °C)	
	bio3 (isothermality (bio2/bio7) ×100; %)	
	bio4 (temperature seasonality (standard deviation ×100); °C)	
	bio5 (maximum temperature of warmest month; °C)	
	bio6 (minimum temperature of coldest month; °C)	
	bio7 (temperature annual range (bio5-bio6); °C)	
	bio8 (mean temperature of wettest quarter; °C)	
	bio9 (mean temperature of driest quarter; °C)	
	bio10 (mean temperature of warmest quarter; °C)	
	bio11 (mean temperature of coldest quarter; °C)	
	bio12 (annual precipitation; mm)	
	bio13 (precipitation of wettest month; mm)	
	bio14 (precipitation of driest month; mm)	
	bio15 (precipitation seasonality; mm)	
	bio16 (precipitation of wettest quarter; mm)	
	bio17 (precipitation of driest quarter; mm)	
	bio18 (precipitation of warmest quarter; mm)	
	bio19 (precipitation of coldest quarter; mm)	
	DD > 0°C (annual cumulative degree days >0°C)	
SNODAS (1 km)	snow cover (number of days with snow cover from December to March; days)	None
	snow depth (cumulative snow depth from December to March; mm)	

*Land cover—* Land cover data were from the the Spatialized CAnadian National Forest Inventory (SCANFI) data product created in 2020 at a 30 m spatial resolution [42]. These data are derived from composite Landsat satellite imagery and classify the Canadian landscape into eight categories: bryoid (bryophytes, non-vascular plants, e.g., mosses), herbs (herbaceous, vascular plants, e.g., grasses), urban (urban and built-up areas), shrub, broadleaf trees (deciduous trees), needleleaf trees (coniferous trees), and water (Fig 1B). Along with land cover types, vegetation attributes including canopy height, crown closure, and above-ground tree biomass were also included [42].

*Topography—* The mean elevation, slope, and aspect were computed from the Shuttle Radar Topography Mission (SRTM) topographical data, which has a 30 m spatial resolution [43].

*Soil attributes—* The soil data are sourced from SoilGrids, which provides global predictions across seven standard depths at a 250 m spatial resolution [44]. These predictions rely on a combination of remote sensing-derived soil variables and ML techniques, predominantly random forest and gradient boosting. The dataset includes six variables including soil organic carbon (SOC) content, soil pH, bulk density (BD) of the fine Earth fraction (< 2 mm), as well as the percentages of sand, silt, and clay. All these features are computed for the top five cm of the soil.

*Bioclimatic—* To explore the impacts of temperature and precipitation, we derived bioclimatic predictor variables from the Daymet dataset, which provides daily meteorological data at a 1 km spatial resolution across North America since 1980 CE. Daymet integrates data from weather stations and other supporting sources through interpolation methods [45]. Using this dataset, we calculated a set of 19 raster-based predictor variables, based on annual data for each year, following the definitions of the WorldClim [46]. Additionally, we computed an annual DD > 0°C, using daily maximum temperature data from Daymet (Fig 1C). In addition to temperature and precipitation, two features relating to snow cover and snow depth were included. These predictor variables represent the total number of days with snow cover and the cumulative snow depth from December to March, sourced from the SNODAS dataset [47].

To account for variation in spatial scales of environmental factors that may associate with tick population ecology, we calculated focal statistics for each predictor variable across multiple spatial scales. Specifically, we assessed for five spatial scales by defining zones around each tick sampling site, with radii ranging from 500 to 2500 m in 500-m increments. As described in the Modelling Approach, we then identified the scale at which the predictive performance in our models was maximized. Except for land cover types, calculated as the proportion of each type, all other predictor variables were summarized as their mean value within the buffered area. Temporally dynamic predictor variables (i.e., bioclimatic predictors, as opposed to static predictors for land cover, elevation and soil attributes) were averaged over two-year periods. This approach reflects the requirement for at least two years of suitable conditions for ticks to complete their life cycles and establish endemic populations. The averaging was performed after the bioclimatic predictor variables were calculated for each year by taking the mean of the current year and the preceding year.

### Overview of modelling approach

In brief, the workflow of the modelling approach was to process two data streams (EO and tick surveillance data), train and assess performance of multiple ML models, and then apply the final selected predictive model to map the probability of an established tick population (Fig 2). Data processing included calculating focal statistics for the predictor variables at specific buffer radii located at the surveillance sites, assessing for multicollinearity among predictor variables, and adjusting tick abundance for seasonal variation given different sampling dates. Using the processed data, a range of ML models were trained, validated and evaluated. The models were tree-based methods (random forest (RF), extreme gradient boosting (XGBoost), gradient boosting (GB), AdaBoost), support vector machines (SVM), multi-layer perceptron (MLP), K-nearest neighbours (KNN), and linear models (logistic regression (LR), ridge classifier, linear discriminant analysis (LDA)). During model training, the optimal parameters and the best set of predictor variables for each model were identified using Quebec surveillance data from 2014 to 2021. External validation was conducted to evaluate the models using unseen data from Quebec (2022–2023). The entire modeling process was repeated across multiple spatial resolutions by applying buffer radii of 500, 1 000, 1 500, 2 000, and 2 500 m around each site. This allowed us to identify the spatial scale at which predictor variables optimized model performance for predicting tick population establishment for each ML model, and select the best-performing model as the final predictive model.

The final predictive model was then assessed for spatial transferability by cross-regional external validation using unseen data from Ontario (2015–2018). The same model was also examined, given the Quebec training data (2014–2021), for the relative importance of the selected predictor variables to assess their contributions to the final predicted outcome. Finally, we used the model to map the probability of tick population establishment for year 2022 over southern Quebec and Ontario at 250 m resolution.

### Data processing

The surveillance data at each sampling site contained the sampling date, geographic coordinates, and the number of detected ticks for life stages (larvae, nymphs, adults). Since surveillance occurred from late spring to early fall, tick life-stage abundances were adjusted to account for seasonal variation. Previously established associations between season and life-stage abundances in the same region were used to estimate the expected abundances of larvae, nymphs and adults, per site, for the month of July [48]. This adjustment mitigated the influence of month-to-month variations in surveillance timing when weighing their contribution during model training. By aligning the abundance estimates to a common point in the season, the adjustment also helped ensure that model predictions reflected spatial differences in environmental conditions rather than phenological variations. This was also important given that the predictor variables represented fixed time periods.

A binary outcome variable was used to indicate the potential presence or absence of established tick populations at the sampling sites. Sites were classified as “presence” (1) if at least one questing tick was detected at either the larval, nymphal or adult life stage, and as “absence” (0) if no ticks were detected. Although various indicators of established populations using active surveillance data exist, no single definition is established across scientific literature [49]. Given the low sensitivity but high specificity of this type of data, and to ensure good predictive power for our model, we chose this permissive threshold [50].

For the presence sites, tick density was used as a weighting factor during the model training to reflect variation in the proportion of life stages. To calculate the density, the seasonally-adjusted counts of larvae, nymphs, and adults were first multiplied by 1, 10 and 52, respectively. This weighting reflects the expected proportion of life stages for feeding larvae, nymphs and adults in populations at equilibrium, based on a modelling study parameterized with data from a similar region [17]. The lower weighting of the younger life stages also accounted for the biological trait that immature ticks, particularly larvae, are commonly found in clumps during drag sampling due to simultaneous hatching of thousands of eggs which produces hundreds of larvae with limited mobility [51,52]. For each sampling site, tick density was then calculated by summing the adjusted values and dividing by the sampling area. This life-stage ratio adjustment served to accentuate biologically meaningful differences between sites. Sites with adult ticks received substantially higher weights, reinforcing the model’s ability to learn associations between predictor variables and ecological conditions that were more favourable for sustaining tick populations. Moreover, a logarithmic transformation of tick density was used to mitigate the skewness of the weights and to improve the model convergence while preserving the relative differences. The inverse of DD > 0°C was also used to weigh the absence sites given the effect of temperature on tick development and their capacity to establish viable populations [53]. As a result, tick-positive sites with higher densities were given greater weights during training, and tick-negative sites with lower DD > 0°C values were also prioritized with higher weights compared to others in their class. Finally, to balance the influence of absence and presence sites, the total weights for both groups were normalized to achieve equal values.

To increase the interpretability and performance of the models, the presence of multicollinearity was tested using the variance inflation factor (VIF). The VIF process involves regressing each predictor variable against the remaining variables and eliminating the values above a cutoff limit, typically between three and 10 [54]. Before applying VIF, each predictor variable was normalized by its mean and standard deviation (i.e., z-score normalization). The normality of the predictor variables was assessed using the Shapiro-Wilk test, and non-normally distributed predictor variables were approximately adjusted through logarithmic, square-root, and Box-Cox transformations. The identification of correlated predictor variables was performed using the recursive elimination strategy. In each iteration, the predictor with the highest VIF score was removed, followed by recalculating the VIF scores for the remaining predictors. This procedure continued until all the features had a VIF score less than five.

After performing recursive elimination, the excluded predictor variables were grouped into clusters based on thematic categories: land cover, soil, topographical and bioclimatic. This clustering was necessary because many predictor variables within each category were correlated and could convey similar types of information. To retain the most informative predictors from among the excluded features, we assessed their point-biserial correlation coefficients with the binary outcome for tick population establishment. For each cluster, we selected the excluded predictors with the strongest positive and negative associations (i.e., significant correlation coefficients > 0.2 or <−0.2 using p-values < 0.05) to be reintegrated into the feature set. This allowed us to preserve potentially important but correlated predictor variables that might otherwise have been eliminated solely due to multicollinearity and ensure a better balance between reducing redundancy and keeping explanatory power.

### Model optimization and predictor variable selection

The model parameters and their best set of predictor variables were determined using the Quebec surveillance data from 2014 to 2021. Site weights were assigned to the outcome variable to mitigate the class imbalance problem and enhance the generalization of the model [55]. K-fold cross-validation was used to assess model performance. To prevent overfitting to a single or atypical year, we divided our data into subsets corresponding to each collection year, resulting in K = 8 folds for the eight years from 2014 to 2021. First, the optimal hyperparameters were determined by using the grid search technique (S1 Table in S1 File). To identify the optimal set of predictor variables, a backward feature elimination process was applied repeatedly. For each possible number of features, the most frequently selected predictor variables across all the folds were identified and model performance was evaluated using these agreed-upon features in cross-validation. Finally, the feature set that produced the highest overall predictive performance was kept for final model development. This process was repeated for all the models to determine the optimal set of predictor variables for each model.

### Model performance, evaluation and predictor contributions

To assess the predictive accuracy of the models and select the final predictive model, we used data from Quebec (2022–2023) which were not involved in the training process. The model performance metrics were sensitivity, specificity, and F1-score (Equations 1–4), along with Area Under the Receiver Operating Characteristic Curve (ROC AUC) and Cohen’s Kappa. These metrics are calculated based on True Positives (TP), False Positives (FP), False Negatives (FN), and True Negatives (TN). Sensitivity quantifies the proportion of actual positive sites correctly identified, while specificity accounts for the ability of the model to correctly identify negative sites. The F1-score combines sensitivity and precision into a single value, and helps interpret model performance in scenarios with imbalanced classes. Cohen’s Kappa measured the agreement between predicted and observed sites, while accounting for chance agreement. The ROC AUC was used to summarize model performance over a range of classification thresholds.


$$Precision=\frac{TP}{TP+FP}$$ (1)

$$Sensitivity=\frac{TP}{TP+FN}$$ (2)

$$Specificity=\frac{TN}{TN+FP}$$ (3)

$$\begin{array}{c} F1\;\text{-}\;score=\frac{2\;(Precision)(Sensitivity)}{Precision+Sensitivity\;} \end{array}$$ (4)

We also assessed for global and local spatial autocorrelation in the residuals of the models applied to the Quebec data (2022–2023). Spatial autocorrelation can result from sampling biases towards certain locations where predictor variables fail to fully capture spatial similarity, or from unmeasured or missing spatially structured variables. Failing to account for spatial autocorrelation reduces the ability of the ML models to generalize to other areas, and can overestimate the importance of the predictor variables and lead to overfitting. Global spatial autocorrelation was assessed using the Moran’s index (Moran’s I) [56], while local spatial autocorrelation was assessed using Local Indicators of Spatial Association (LISA) which measure Moran’s I at a local level [57]. Under both methods, the null hypothesis is that the data points are spatially independent, and the tests assume normally distributed data. We used LISA to assess for spatial autocorrelation between each site and its five nearest neighbouring sites. That result is reported as the proportion of sites with non-clustered residuals, i.e., where no statistically significant spatial autocorrelation was detected (p-values > 0.05). To ensure that each spatial location contributed only once to the calculation of Moran’s I and LISA, one year of site data was randomly excluded if a site was sampled in both 2022 and 2023.

In the final selected predictive model, given the performance metrics against the unseen Quebec data (2022–2023), the importance of the selected variables was determined using the TreeExplainer method [58]. This method which originates from game theory, represent the contribution of each feature to the prediction at both global and local levels based on SHapley Additive exPlanation (SHAP) values [58]. The SHAP values assigned to each feature indicate its importance and reflect the change in the expected model predictions due to that feature. The ability of the final selected predictive model to spatially generalize to another region was also evaluated. Here we used the Ontario surveillance data (2015–2018), which had been excluded during the training phase, and calculated performance metrics for sensitivity, specificity and ROC AUC.

A map of the predicted probability of tick population establishment in Quebec and Ontario was created for year 2022 at a 250 m resolution. We used this resolution to balance spatial detail with computational efficiency, for deriving the predictor variables from their original resolution to 250 m. The predictor variables, retained in the final selected predictive model, were first projected to the Lambert Conformal Conic coordinate system. For each predictor variable, focal statistics were calculated at their original resolution using neighbourhoods centred on each pixel and including only pixels whose centres fell within the specified radius (i.e., 500, 1 000, 1 500, 2 000 or 2 500 m). For continuous predictors, the focal statistic at a pixel was the mean value within the radius. The exception was the SCANFI land cover data because pixel values were categorical land cover types. In this case, the focal statistic at a pixel was the proportion of a land cover type with the radius, and separate layers were created for each land cover type. After calculating focal statistics at the native resolutions, the predictor variables were resampled to a 250 m resolution, except for the SoilGrids data because these data were already at 250 m. For the resampling process, the finer-scale SCANFI data were downscaled as the mean pixel values falling within the 250 m grid, for each 250 m pixel. The coarser predictors at 1-km (e.g., Daymet, SNODAS) were upscaled to 250 m using nearest-neighbour interpolation. The resulting resampling process also spatially aligned the layers and these layers were then used by the model to estimate the probability of tick population establishment for each pixel.

To assess the reliability of model predictions, an uncertainty quantification (UQ) map was generated using a bootstrap resampling approach. Multiple bootstrap samples (500 iterations) were created from the training data with each sample having the same size of the original data but generated through sampling with replacement to introduce variability. This resampling method ensures the model is well-trained in each iteration and maintains the full dataset structure. This lowers the chance of instability or missing important patterns, and allows the method to capture significant variability in predictions while avoiding excessive noise from small subsets. For each bootstrap sample, the models were trained and model predictions were obtained for each pixel in the study area. Uncertainty for each pixel was calculated as the ratio of the interquartile range (i.e., the difference between 75th and 25th percentiles) to the median.

## Results

### Multicollinearity analysis

**Fig 3:**
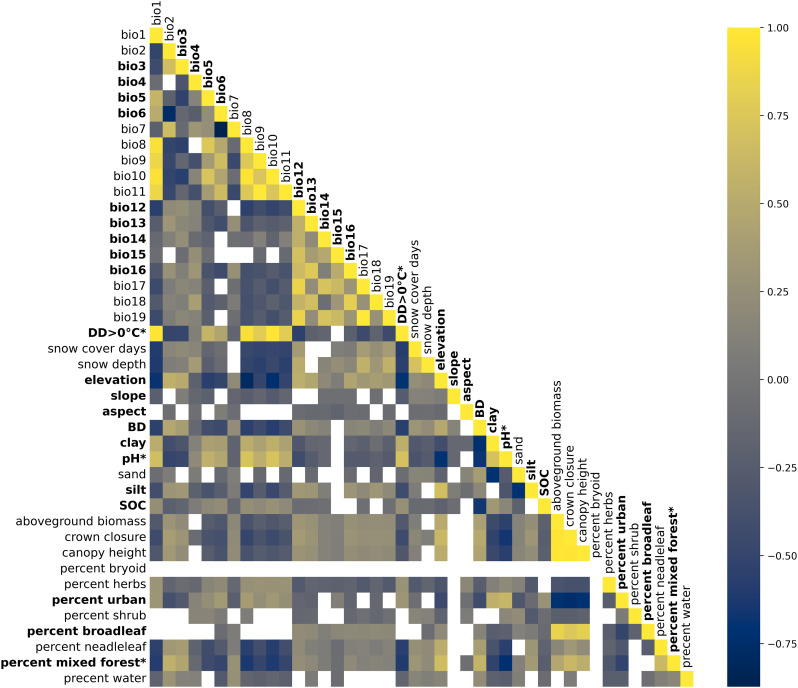
Pearson correlations among the candidate predictor variables, calculated at surveillance sites using a 1 000-m radius buffer. White cells indicate non-significant correlations (p-value > 0.05). Bolded variables were retained for model training. Asterisks indicate predictor variables that were initially excluded during the VIF analysis but subsequently reinstated as based on point-biserial correlation results.

Multicollinearity analysis was conducted as part of the assessment of predictor variables across different spatial scales. The results were largely consistent across buffer radii, with the exception of a few radii where percent water and percent shrub were retained in the final predictor variable set due to reduced collinearity at those scales (S1–S4 Figs in S1 File). The correlation matrix revealed significant relationships among the predictor variables in the best-performing spatial scale (1 000-m radius buffer; Fig 3). The bioclimatic variables (bio1–19) generally had strong inter-correlations, which is expected given that these variables often capture related aspects of climate. For instance, DD > 0°C was strongly correlated with temperature-related variables such as bio1 (r = 0.93; annual mean temperature) and bio8 to bio11 (r = 0.81–0.92; bio8, mean temperature of wettest quarter; bio9, mean temperature of driest quarter; bio10, mean temperature of warmest quarter; bio11, mean temperature of coldest quarter). These correlations support using DD > 0°C as an indicator of warmer climates with longer growing seasons. There was also a notable negative correlation between DD > 0°C and snow cover days (r = −0.58), snow depth (r = −0.62), and elevation (r = −0.70). Elevation also had strong negative correlations with bio1 (r = −0.68; annual mean temperature), bio5 (r = −0.55; maximum temperature of warmest month), and bio6 (r = −0.52; minimum temperature of coldest month), reflecting the decrease in temperature with elevation. BD was correlated negatively with bio1 (r = −0.53; annual mean temperature), and positively with bio2 (r = 0.47; mean diurnal range) and elevation (r = 0.53), suggesting that denser soils were found in cooler and higher areas. Overall, the correlation matrix highlighted interrelationships among environmental factors through correlations among these variables, and the need to address multicollinearity to avoid redundancy and misleading variable effects.

The multicollinearity check using VIF analysis of the predictor variables and their partial reintegration using point-biserial correlation, reduced the set of 42 candidates variables to 21 (Fig 3 bold variables). The excluded variables had inter-categorical correlations, as found with bioclimatic and snow-related predictors, and with vegetation-related predictors (i.e., crown closure, aboveground biomass, percent broadleaf).

### Predictive performance of models

Performance of the ML models was generally highest at the 1 000-m radius, given validation against the unseen Quebec surveillance data from 2022 to 2023 (Tables 2, S2–S6 in S1 File). In general, ensemble tree-based models and SVM were the highest performing models. XGBoost and SVM had strong sensitivity (0.83 and 0.86, respectively), indicating their effectiveness at identifying true positives. However, the relatively lower specificity of SVM suggested a trade-off in misclassifying some negative cases. GB had balanced sensitivity and specificity with a robust F1-score of 0.71. In contrast, models such as MLP and KNN exhibited low ROC AUC and poor Kappa values, indicating less reliable performance for this dataset. The results of the internal cross-validation during the training also showed that XGBoost and GB had lower standard deviations across all accuracy metrics, suggesting stable performance across years (S7 and S8 Tables in S1 File). In contrast, models such as MLP and LDA showed higher variance, meaning their yearly performance was more inconsistent or sensitive to temporal changes or sample composition.

**Table 2:** Performance of ML models for estimating tick population establishment at Quebec surveillance sites from 2022-2023, using predictor variables calculated at the surveillance sites using a 1 000-m radius buffer. Models are ordered by ROC AUC.

Model	Kappa	Sensitivity	Specificity	F1-score	ROC AUC	Moran’s I (p-value)	% Sites with Non-Clustered Residuals
XGBoost	0.53	0.83	0.71	0.78	0.77	−0.017 (0.06)	81.2
AdaBoost	0.52	0.67	0.86	0.74	0.76	0.068 (<0.01)	84.0
SVM	0.43	0.86	0.58	0.75	0.72	0.073(<0.01)	76.8
RF	0.44	0.63	0.81	0.69	0.72	0.021 (0.03)	84.0
GB	0.42	0.72	0.71	0.71	0.72	0.08 (0.09)	81.6
LR	0.37	0.56	0.88	0.67	0.72	−0.011 (0.45)	76.0
Ridge Classifier	0.39	0.77	0.62	0.72	0.70	0.078 (<0.01)	72.8
LDA	0.45	0.57	0.88	0.73	0.67	0.019 (0.03)	91.2
KNN	0.28	0.58	0.70	0.62	0.64	0.049 (<0.01)	80.8
MLP	0.26	0.67	0.61	0.56	0.64	0.102 (<0.01)	55.2

The spatial autocorrelation of residuals, measured by Moran’s I, was generally weak across all the models, with values ranging from −0.017 to 0.102. Among them, some models such as XGBoost, GB, and LR showed non-significant spatial autocorrelation, suggesting more reliable predictions without spatial bias in the residuals. Others, such as MLP, SVM, and Ridge Classifier, showed significant but still relatively low levels of spatial clustering which could undermine the model’s reliability. Overall, we selected XGBoost as the top model for predicting tick population establishment by balancing sensitivity and specificity, and having spatial independence as measured globally and as found for 81.2% of the sites (Table 2).

### Predictor variables and SHAP analysis

The final predictive model (XGBoost) contained a broad range of predictor variables, including bioclimatic, soil, and land cover types (Table 3). The diversity in predictor variable selection across the models reflected varying complexity and predictive power of each algorithm, but the consistent inclusion of DD > 0°C suggested its critical role in tick population establishment. Among the other models, frequently included predictor variables were pH, percent broadleaf, percent urban, bio5 (maximum temperature of warmest month; °C), silt, clay, percent mixed forest, elevation, and slope.

**Table 3:** List of predictor variables included in the final models, calculated at the surveillance sites using a 1 000-meter radius buffer.

Feature	XGBoost	AdaBoost	SVM	RF	GB	LR	Ridge Classifier	LDA	KNN	MLP
DD > 0°C	✓	✓	✓	✓	✓	✓	✓	✓	✓	✓
pH	✓	✓	✓	✓	✓	✓	✓	✓	✓	
percent broadleaf	✓	✓	✓	✓	✓	✓	✓	✓	✓	
percent urban	✓	✓	✓		✓	✓	✓	✓	✓	
bio5 (maximum temperature of warmest month; °C)	✓	✓	✓	✓	✓	✓	✓	✓	✓	
silt	✓	✓	✓		✓	✓	✓	✓	✓	
clay	✓	✓		✓	✓	✓	✓	✓	✓	
percent mixed forest	✓	✓	✓	✓	✓	✓		✓	✓	
elevation			✓		✓	✓	✓	✓	✓	✓
slope		✓	✓		✓	✓	✓	✓	✓	
bio15 (precipitation seasonality; mm)		✓	✓			✓	✓	✓	✓	
bio6 (minimum temperature of coldest month; °C)		✓	✓			✓	✓	✓	✓	
SOC	✓					✓	✓	✓	✓	
bio12 (annual precipitation; mm)			✓			✓	✓	✓	✓	
BD (bulk density)			✓			✓	✓	✓	✓	
bio13 (precipitation of wettest month; mm)			✓			✓	✓	✓	✓	
aspect		✓	✓				✓		✓	
bio3 (Isothermality (BIO2/BIO7) ×100; %)			✓			✓			✓	
bio4 (temperature seasonality (standard deviation ×100); °C)			✓			✓			✓	
bio14 (precipitation of the driest month; mm)			✓			✓	✓			
bio16 (precipitation of wettest quarter; mm)						✓	✓		✓	

**Fig 4:**
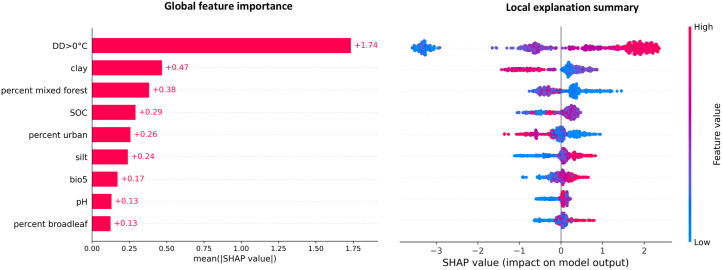
Global and local feature (predictor variable) explanations from the XGBoost trained with Quebec data (2014–2021). Left: bar chart showing the global importance of each feature, measured as the mean absolute SHAP value across all observations. Higher values indicate greater overall influence on the model’s predictions. Right: The local explanation summary plot indicates how each feature observation contributes to the model’s predictions. Each dot represents a site, with colour indicating the feature value (red = high, blue = low). Dot position along the x-axis is the SHAP value, showing how much that feature shifts the model’s prediction from the baseline on a log-odds scale, with positive values increasing the prediction and negative values decreasing the prediction. The baseline prediction (the model’s average output) was a log-odds of approximately −0.212, corresponding to a probability of about 0.44. For a single feature, predicted log-odds for a site is calculated by adding that feature’s SHAP value to the baseline. For example, a high DD > 0°C value contributing a SHAP value of +2.2 would increase the predicted probability from the baseline of 0.44 to 0.88 as follows: log-odds = Baseline + SHAP_DD > 0 = −0.212 + 2.2 = 1.988 and the final probability, p, would be p = 1/ (1 + e^(−1.988)) ≈ 0.88.

The SHAP analysis of the final predictive model (XGBoost), trained on Quebec data from 2014 to 2021, indicated that the main drivers influencing model predictions were warmer temperatures (DD > 0°C and bio5, maximum temperature of warmest month), silty soil (lower clay content) with slightly higher than average SOC and pH, and land cover types that contained broadleaf forests (percent mixed forest, percent broadleaf) and less urban areas. The global feature importances showed that DD > 0°C had a dominate impact on the prediction among the predictor variables (Fig 4). The local SHAP summary further revealed that high values of predictor variables DD > 0°C, silt, bio5, and percent broadleaf, consistently pushed model prediction toward a higher probability of tick population establishment; while some predictor variables, such as percent urban and percent mixed forest, negatively impacted tick population establishment.

### Maps of predicted probability and uncertainty

**Fig 5:**
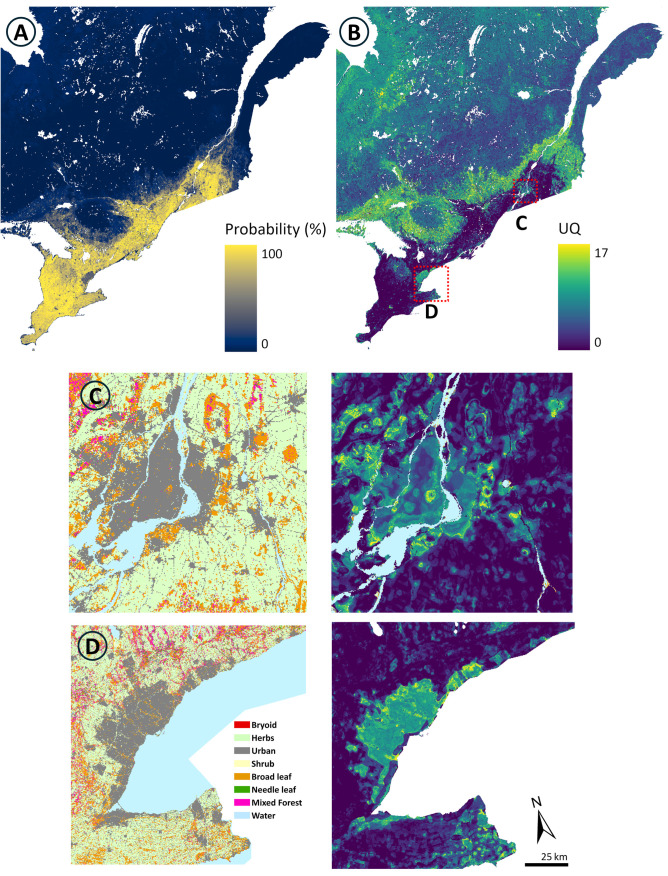
XGBoost model output for 2022 at 250 m resolution. A) Predicted probability map of the established tick population, and B) associated uncertainty quantification (UQ). Shown in C) and D) are zoomed-in sections of the UQ map with corresponding land cover maps, illustrating areas of high uncertainty in Montreal, Quebec and Toronto, Ontario, respectively.

The map of the predicted probability of tick population establishment illustrates that most of southern Ontario and Quebec have a high probability of containing established tick populations (Fig 5A). Cross-regional external validation results had mixed performance, highlighting both strengths and limitations in the ability of the model to extrapolate predictions to new regions. The model showed high sensitivity (0.80) in detecting locations with established tick populations, but low specificity (0.32), which was further reflected by the trade-off between sensitivity and specificity, as shown from the ROC AUC score of 0.56. Model results also indicate high uncertainty in the most urban dense areas of the probability map, corresponding to the metropolitan areas of Montreal (Fig 5C) and Toronto (Fig 5D).

## Discussion

This study developed a predictive model for blacklegged tick, *Ixodes scapularis*, population establishment using bioclimatic and habitat variables mostly derived from EO data. The primary objective was to identify the best-performing model and predictor variables that produced accurate predictions for Quebec, and secondarily, whether the model could be scaled to Ontario, Canada. The results from our analysis highlighted the effectiveness of ensemble tree-based models, with XGBoost as the top performing model, in predicting tick population establishment. Predictor variables derived from a 1 000 m radius around the surveillance sites had the highest model performance when compared to variables created at the other assessed radii.

This study underscored challenges posed by the inherent complexity of environmental data for ecological modeling. A central issue is balancing the inclusion of informative environmental variables and avoiding overfitting, to ensure model accuracy and transferability [56]. While ML algorithms can handle large datasets, redundant variables can obscure meaningful ecological relationships. VIF analyses addressed multicollinearity in predictor variables relating to interrelated aspects of the environment to develop more robust and interpretable models. Additionally, assessing spatial autocorrelation in model residuals helped identify models that were less subject to spatial biases and better suited for generalizing to new regions or datasets. Among the models, LR retained the most predictors (19), while MLP used only elevation and DD > 0°C. Although DD > 0°C is a key factor for tick population establishment, the MLP model appears too limited for capturing broader ecological drivers relating to tick habitat and host availability. The high and statistically significant spatial autocorrelation in the MLP residuals further suggested that important environmental predictors were missing, and that predictions may have been influenced by spatial clustering rather than true ecological patterns. These findings underscore the importance of incorporating a diverse range of plausible environmental variables and developing multiple models to reduce spatial bias and better capture factors predicting tick population establishment.

Previous studies have reported a range of bioclimatic variables affecting the tick species distribution, of which, inconsistencies in these variables may relate to differences in data availability, modelling approaches or the specific ecology of the study area [59,60]. In this study, DD > 0°C was identified as the key predictor of tick population establishment, which is consistent with previous studies in this region [43,61,62]. In Canada, where the northern boundary of the tick species distribution now occurs, this result reflects the biological requirement for sufficient warmth to enable ticks to complete their life cycle [17]. In areas with temperature conditions above the DD > 0°C threshold, other ecological factors affecting tick ecology may also determine the presence and abundance of *I. scapularis*. The warmer temperature correlated with less severe winter conditions may increase the density of key hosts, such as white-tailed deer, which are the reproductive hosts for the ticks [63]. Moreover, mild winters favour survival of small mammals such as white-footed mice which are an important host for ticks during larval stages [64]. The snow variables, snow cover days and snow depth, were not retained in the models. While snow cover may provide insulation protecting ticks from extreme cold, reducing overwinter mortality [26,27,65], the benefits of this effect may be outweighed by other factors in our study area, such as ticks having sufficient warmth to complete their life cycle and reproduce.

In addition to climate-related factors, soil properties were also identified to influence the probability of tick population establishment. SOC, silt content, and pH serve as proxies for soil moisture retention and nutrient availability, and higher values had positive associations with tick population establishment. Higher levels of SOC can help in retaining moisture and creating microhabitats that are more favourable for ticks [32]. Soil texture influences the extent of drainage. Silt particles, with a size between sand and clay, provide a balanced capacity to retain moisture and nutrients while still allowing for moderate drainage. Previous studies in this region of Canada have identified a greater likelihood of *I. scapularis* population establishment [62], or lower tick mortality [66], where soils have more clay and less sand. Also, the pH of soil influences nutrient availability by altering the chemical forms and solubility of nutrients within the soil. At the sampling sites, pH levels ranged from 4 to 6.2 which indicates slightly to moderately acidic conditions. The local SHAP analysis revealed that lower pH values had a greater influence on reducing the probability of tick population establishment compared to higher pH levels. This observation aligns with existing knowledge that acidic soils (lower pH) can limit nutrient availability [67]. This may explain why some coniferous forests which create more acidic soil conditions, through the decomposition of their leaf litter, may be less suitable tick habitat [68].

Land cover attributes, such as the percentage of broadleaf trees, were also important predictors of tick population establishment. Broadleaf forests provide a stable and humid microclimate that supports tick survival, with leaf litter being particularly important during immature stages [22]. These forests also provide habitat for host species important to ticks, including small mammals and white-tailed deer. In contrast, our models indicated that urban areas had a negative impact on tick population establishment. This is consistent with urban areas having few green spaces providing suitable microhabitats for tick survival. However, forested urban riverain zones and suburban areas in proximity to natural habitats, where green spaces are large enough to support small mammals and white-tailed deer, can still support tick populations [69]. The higher probabilities of tick populations coupled with high uncertainty from our model results in the densely populated urban areas of our study area may reflect the spatial resolution of the analysis being too coarse to capture the presence of green spaces which can support tick populations. Future studies could assess the value of using higher resolution and raw sensor EO data to improve spatial accuracy and better capture habitat heterogeneity. Furthermore, limited sampling data in urban areas may have contributed to lower model performance, highlighting the need for targeted tick surveillance in these areas to improve model reliability by addressing data gaps.

Limitations to ENMs are inherent to the data-driven approach. Predictive accuracy depends on data quantity and quality, and the appropriateness of methods used to derive predictor variables relevant to tick ecology [70]. In our study area, we were not limited by the spatio-temporal distribution of available geospatial data products for creating predictor variables. However, uncertainties in the geospatial data products, such as misclassifications or low accuracy in certain regions, can compromise model prediction reliability. Shifting towards the use of raw sensor EO data could mitigate this issue and enhance model performance by providing more accurate and consistent input data [71]. With regards to surveillance data, obtaining a homogeneous spatio-temporal distribution of these data is often impractical given resource constraints or is at conflict with the goals of surveillance (e.g., monitoring spread into new areas). Though in our study, the surveillance data had wide spatio-temporal coverage, and we could build models with high predictive accuracy. The uncertainty analysis further strengthened our approach by identifying areas where model performance was weaker, to help guide interpretation and highlight regions for future data collection.

Study limitations also concern the ecological relevance of the predictor variables to the outcome of interest. Firstly, we used focal statistics, such as neighbourhood averaging, to represent local environmental effects on tick population establishment at the sampling site. While this method is effective to some extent, this approach likely smooths over fine-scale spatial heterogeneity and disrupts spatial dependencies that are important for tick ecology. Future models should aim to incorporate more advanced spatial structure techniques, such as convolution deep learning methods that better capture the complexity of ecological interactions and spatial dependencies [72]. Secondly, there is also a lack of understanding regarding the spatial scales that are appropriate for deriving predictor variables [60]. Presumably some predictor variables impact tick population ecology at larger spatial scales than other predictor variables [60], for example, widespread coverage of sufficient DD > 0°C over our study area compared to finer-scale and more abrupt changes in land cover types. Predictor variables may also represent different ecological processes at different spatial scales (e.g., large scale forest cover providing deer habitat, an important host for ticks, versus small-scale variations in leaf litter, affecting tick survival differently along the forest floor). Although we did not assess for differences in spatial scale among the predictor variables within models (all predictor variables within a given model were derived from the same spatial scale), we did compare models that differed in the spatial scale at which predictor variables were derived and found that the model-wide spatial scale of predictors influenced model performance, as has been reported in other studies [29,48]. Further study is needed to understand how the spatial scale of predictor variables represent ecological processes associated with the outcome of interest (e.g., population presence/absence, nymph density). This also includes consideration of temporal scales of predictor variables, and recognition that such explorations must be grounded in the spatio-temporal scales over which the surveillance strategy can reliably measure the outcome.

We were motivated to develop a modelling approach that can support public health management of zoonotic diseases caused by *I. scapularis*, particularly LD. Though ENM studies increasingly train multiple ML models and evaluate them with several performance metrics, we also considered this approach useful from a public health perspective. High sensitivity is favourable when the priority is to minimize the risk of overlooking or underestimating a threat. Both the XGBoost and SVM models had the highest sensitivities, however, low specificity of the SVM could mislead public health authorities to target efforts in areas with negligible risk. Furthermore, low specificity that resulted when the XGBoost was cross-validated with Ontario data may reflect a timing mismatch. The Ontario surveillance data (2015–2018) was collected during a period of population expansion, whereas the prediction year (2022) may reflect conditions by which some sites had already become established. Consequently, some environmentally suitable sites classified as false negatives may have resulted because tick densities were too low to detect via drag sampling [37] or where rates of importation of ticks into environmentally suitable locations had been too low to initiate reproducing tick populations [6,7].

We also consider our approach of value to public health by using open-access EO data. The resulting approach enables a generation of affordable (in the sense of open-access data), scalable, updatable risk maps for tick population establishment that can inform resource allocation and policy decisions. To facilitate risk communications, the generated maps can be integrated into web and mobile mapping applications so the public and health practitioners can zoom in precisely on locations of interest. While the developed model does not account for pathogen circulation, it is important to note that pathogens typically emerge a few years after tick establishment and will increase disease transmission risk as tick densities and pathogen prevalence grow under suitable conditions. Therefore, our modelling approach could serve as a practical tool for public health to prioritize surveillance and intervention efforts, particularly in the face of climate change and land use changes which continually change risk of vector-borne diseases [73]. By integrating future climate scenario data, the model can estimate the expansion of the established tick population. This, in turn, provides insights into the climatic and habitat factors influencing the distribution of established tick populations and tick-borne disease risk.

## Conclusion

In this study, we developed a ML framework largely based on EO data to estimate the probability of blacklegged tick population establishment in Quebec and Ontario, Canada. Among the tested models and spatial scales for deriving the predictor variables, ensemble models, particularly XGBoost, demonstrated high performance at a 1 000-m radius around surveillance sites. The model was validated through time-based cross-validation, analysis of residual spatial autocorrelation, and evaluated for spatial transferability to Ontario. Model results indicate that key environmental determinants for tick population establishment are DD > 0°C, soil properties such as silt content and soil organic carbon, and broadleaf forest land cover, whereas urban areas appear to limit it. The modelling approach enables creation of high resolution and updatable risk maps to identify areas of established tick populations which can inform public health management of zoonotic diseases from blacklegged ticks.

## Supporting information

S1 File Tables 1–8, Figures 1–4. (DOCX)
