# Supplementary material for: A machine learning framework for estimating the probability of blacklegged tick population establishment in eastern Canada using Earth observation data
Source: PLoS One. 2025 Sep 22;20(9):e0332582. doi: 10.1371/journal.pone.0332582 (PMC12453211; doi:10.1371/journal.pone.0332582)
Supplement: S1 File — (DOCX) [file pone.0332582.s001.docx]

**Supporting Information**

A machine learning framework for estimating the probability of blacklegged tick population establishment in eastern Canada using Earth observation data

Hamid Ghanbari^1,2^, Kevin Siebels^1,2^, Ariane Dumas^1,2^, Emily S. Acheson^1,2^, Catherine Bouchard^1,2,3^, Kirsten Crandall^4^, Patrick A. Leighton^2,3^, Nicholas H. Ogden^1,2,3^, Erin E. Rees^1,2,3*^

^1^ Modelling Hub Division, Applied Public Health Sciences Directorate, Science and Policy Integration Branch, Public Health Agency of Canada, Québec, Canada,

^2^ Groupe de recherche en épidémiologie des zoonoses et santé publique, University of Montréal, Québec, Canada,

^3^ Département de pathologie et microbiologie, Faculté de médecine vétérinaire, Université de Montréal, Québec, Canada,

^4^ Unité maladies infectieuses en communauté, Direction des risques biologiques, Institut national de santé publique du Québec, Québec, Canada

*Corresponding author

E-mail: [erin.rees@phac-aspc.gc.ca](mailto:erin.rees@phac-aspc.gc.ca)

**Table S1.** Hyperparameter ranges used for model optimization during grid search. Ranges and values were selected based on commonly effective defaults and relevant literature.

| Model | Hyperparameter | Range/Options | Notes |
| --- | --- | --- | --- |
| GB | n_estimators | 40 to 200 (step = 10) | Number of trees |
|  | learning_rate | [0.001, 0.01, 0.1, 1, 10] | Step size shrinkage used to prevent overfitting |
|  | max_depth | [2, 3, 5, 7] | Maximum depth of each decision tree |
| RF | n_estimators | 40 to 300 (step = 10) | Number of trees |
|  | max_features | ['sqrt', 'log2'] | Number of features considered at each split |
|  | max_depth | [2, 3, 5, 7] | Maximum depth of each decision tree |
| XGBoost | n_estimators | 40 to 300 (step = 10) | Number of trees |
|  | learning_rate | [0.001, 0.01, 0.05, 0.1, 0.5, 1, 10] | Step size shrinkage used to prevent overfitting |
|  | max_depth | [2, 3, 4, 5, 6, 7] | Maximum depth of each decision tree |
| AdaBoost | n_estimators | 40 to 200 (step = 10) | Number of trees |
|  | learning_rate | [0.001, 0.01, 0.05, 0.1, 0.5, 1, 10] | Step size shrinkage used to prevent overfitting |
| SVM | C | [0.01, 0.1, 1, 10] | Regularization strength |
|  | kernel | ['linear', 'rbf'] | Type of kernel |
|  | gamma | ['scale', 'auto'] | Kernel coefficient |
| MLP | hidden_layer_sizes | [(20,), (50,), (100,), (50, 25), (100, 50), (100, 50, 25)] | Neurons per layer |
|  | activation | ['tanh', 'relu'] | Activation function |
|  | solver | ['adam', 'sgd'] | Optimization algorithm |
|  | alpha | [0.0001, 0.001, 0.01, 0.1, 1.0] | L2 regularization |
|  | learning_rate | ['constant', 'adaptive'] | Learning rate schedule |
| KNN | n_neighbors | [3, 5, 7] | Number of neighbors |
|  | weights | ['uniform', 'distance'] | Weight function |
|  | metric | ['euclidean', 'manhattan', 'minkowski'] | Distance metric |
| LDA | solver | ['lsqr', 'eigen'] | Algorithm used for decomposition |
|  | shrinkage | [None, 'auto', 0.1, 0.5] | Regularization for covariance |
| LR | penalty | ['l1', 'l2'] | Regularization type |
|  | C | [0.1, 1, 10] | Inverse of regularization strength |
|  | solver | ['saga'] | Optimization algorithm |
| Ridge Classifier | alpha | [0.1, 1.0, 10.0] | Regularization strength |
|  | solver | ['svd', 'cholesky', 'lsqr', 'saga'] | Optimization algorithm |


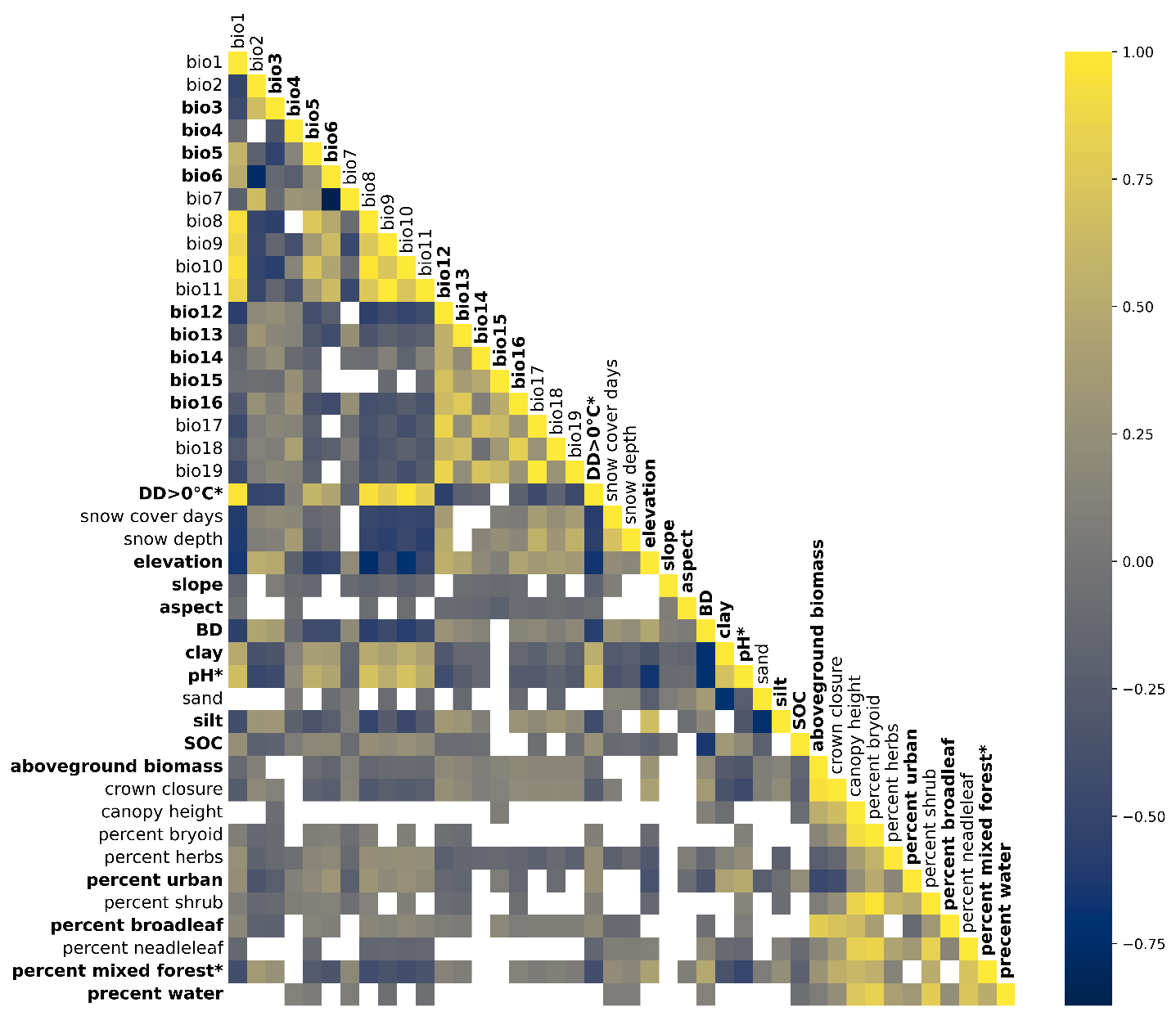


**Fig S1.** Pearson correlations among the candidate predictor variables, calculated at surveillance sites using a 500-meter radius buffer. White cells indicate non-significant correlations (p-value > 0.05). Bolded variables were retained for model training. Asterisks indicate predictor variables that were initially excluded during the VIF analysis but subsequently reinstated as based on point-biserial correlation results.


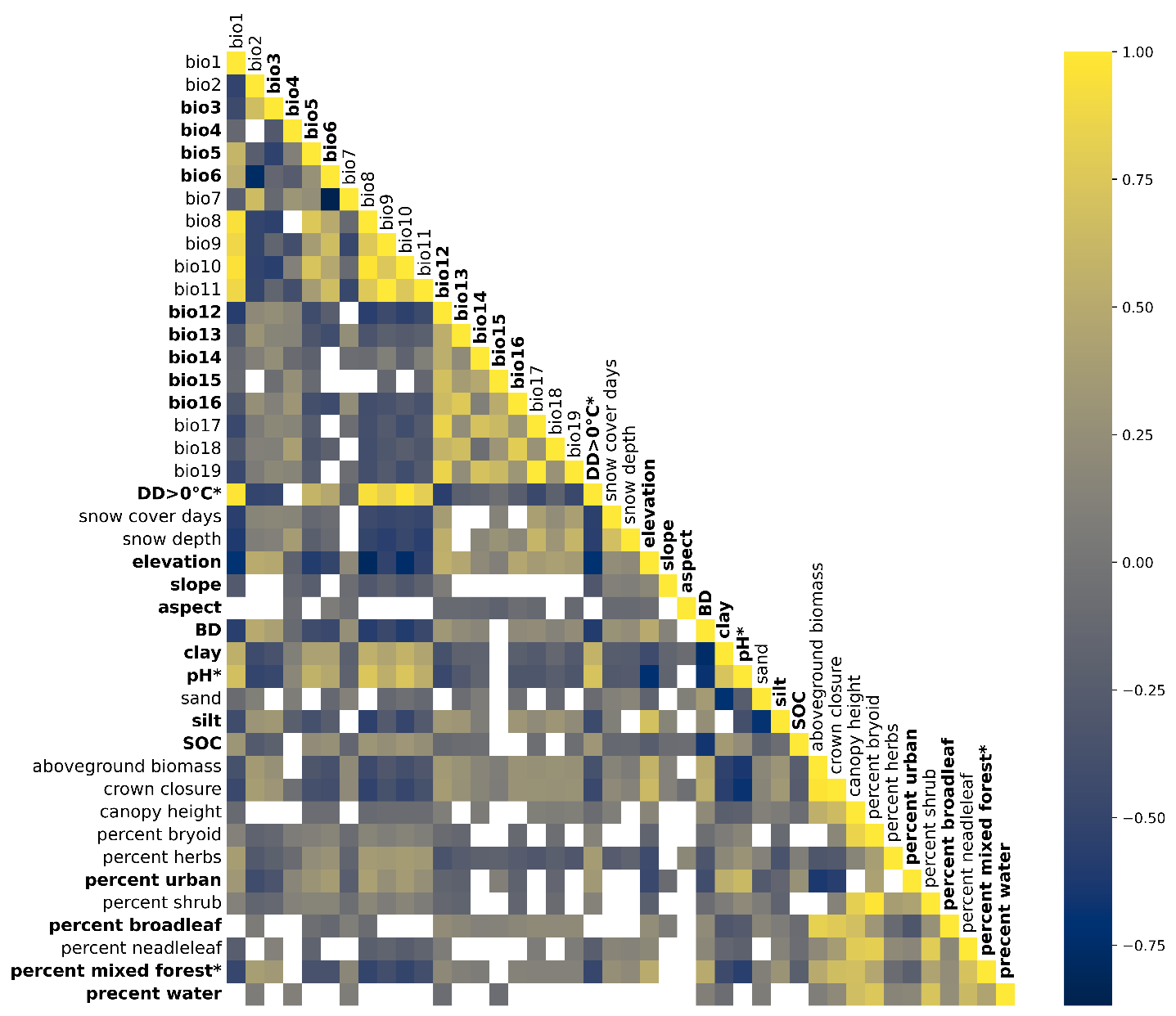


**Fig S2.** Pearson correlations among the candidate predictor variables, calculated at surveillance sites using a 1500-meter radius buffer. White cells indicate non-significant correlations (p-value > 0.05). Bolded variables were retained for model training. Asterisks indicate predictor variables that were initially excluded during the VIF analysis but subsequently reinstated as based on point-biserial correlation results.


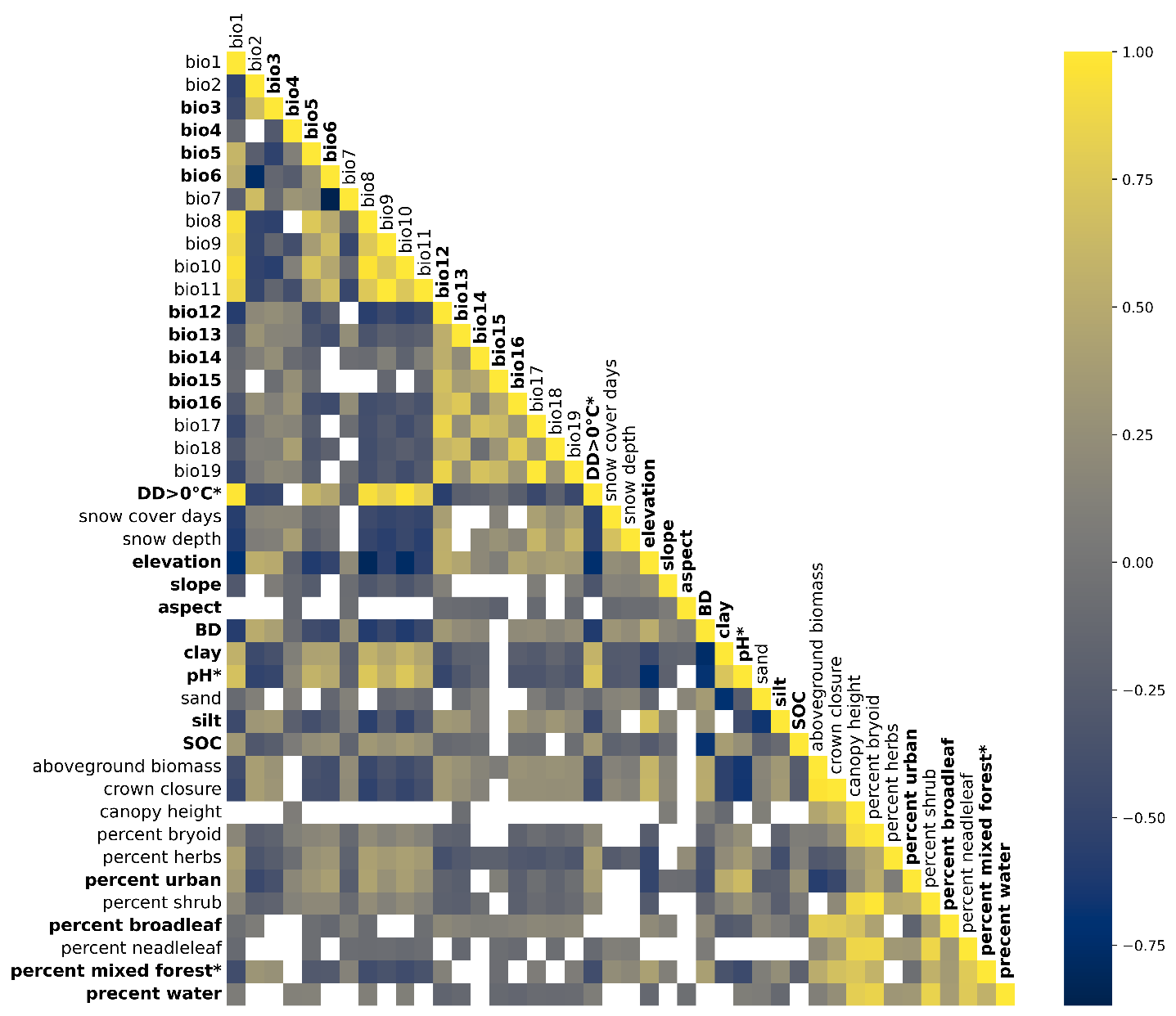


**Fig S3.** Pearson correlations among the candidate predictor variables, calculated at surveillance sites using a 2000-meter radius buffer. White cells indicate non-significant correlations (p-value > 0.05). Bolded variables were retained for model training. Asterisks indicate predictor variables that were initially excluded during the VIF analysis but subsequently reinstated as based on point-biserial correlation results.


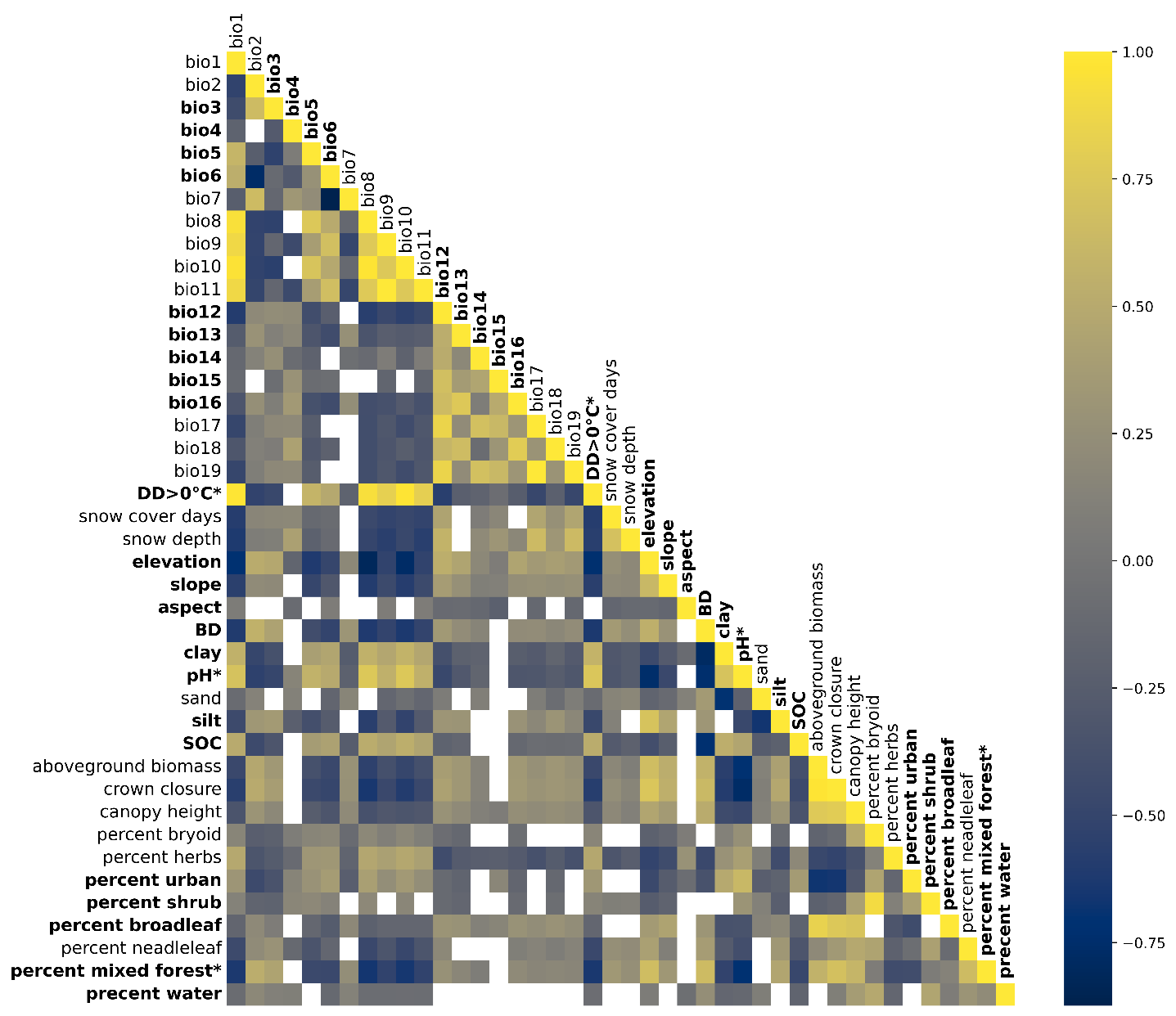


**Fig S4.** Pearson correlations among the candidate predictor variables, calculated at surveillance sites using a 2500-meter radius buffer. White cells indicate non-significant correlations (p-value > 0.05). Bolded variables were retained for model training. Asterisks indicate predictor variables that were initially excluded during the VIF analysis but subsequently reinstated as based on point-biserial correlation results.

**Table S2.** Performance of ML models for tick presence estimation on Quebec surveillance sites from 2022-2023, given predictor variables calculated at the surveillance sites using a 500-meter radius buffer.

| Model | Kappa | Sensitivity | Specificity | F1-score | | ROC AUC | Moran's I  (p-value) | % Sites with Non-Clustered Residuals |
| --- | --- | --- | --- | --- | --- | --- | --- | --- |
| XGBoost | 0.50 | 0.77 | 0.72 | 0.73 | 0.74 | | -0.008 (0.41) | 76.0 |
| AdaBoost | 0.34 | 0.50 | 0.83 | 0.59 | 0.67 | | 0.075 (<0.01) | 77.6 |
| SVM | 0.47 | 0.89 | 0.60 | 0.75 | 0.73 | | 0.075 (<0.01) | 78.4 |
| RF | 0.50 | 0.79 | 0.71 | 0.73 | 0.74 | | 0.029 (0.01) | 80.0 |
| GB | 0.36 | 0.43 | 0.92 | 0.56 | 0.67 | | 0.024 (0.02) | 73.6 |
| LR | 0.29 | 0.69 | 0.61 | 0.64 | 0.65 | | -0.009 (0.4) | 76.0 |
| Ridge Classifier | 0.44 | 0.60 | 0.83 | 0.67 | 0.72 | | 0.094 (<0.01) | 76.0 |
| LDA | 0.50 | 0.73 | 0.79 | 0.72 | 0.72 | | 0.033 (<0.01) | 90.4 |
| KNN | 0.30 | 0.58 | 0.71 | 0.60 | 0.65 | | -0.052 (<0.01) | 84.8 |
| MLP | 0.34 | 0.64 | 0.70 | 0.64 | 0.67 | | 0.058 (<0.01) | 76.8 |
| Average | 0.40 | 0.66 | 0.74 | 0.66 | 0.70 | | — | 78.9 |

**Table S3.** Performance of ML models for tick presence estimation on Quebec surveillance sites from 2022-2023, given predictor variables calculated at the surveillance sites using a 1000-meter radius buffer.

| Model | Kappa | Sensitivity | Specificity | F1-score | ROC AUC | Moran's I  (p-value) | % Sites with Non-Clustered Residuals |
| --- | --- | --- | --- | --- | --- | --- | --- |
| XGBoost | 0.53 | 0.83 | 0.71 | 0.78 | 0.77 | -0.017 (0.06) | 81.2 |
| AdaBoost | 0.52 | 0.67 | 0.86 | 0.74 | 0.76 | 0.068 (<0.01) | 84.0 |
| SVM | 0.43 | 0.86 | 0.58 | 0.75 | 0.72 | 0.073(<0.01) | 76.8 |
| RF | 0.44 | 0.63 | 0.81 | 0.69 | 0.72 | 0.021(0.03) | 84.0 |
| GB | 0.42 | 0.72 | 0.71 | 0.71 | 0.72 | 0.08(0.09) | 81.6 |
| LR | 0.37 | 0.56 | 0.88 | 0.67 | 0.72 | -0.011(0.45) | 76.0 |
| Ridge Classifier | 0.39 | 0.77 | 0.62 | 0.72 | 0.70 | 0.078 (<0.01) | 72.8 |
| LDA | 0.45 | 0.57 | 0.88 | 0.73 | 0.67 | 0.019(0.03) | 91.2 |
| KNN | 0.28 | 0.58 | 0.70 | 0.62 | 0.64 | 0.049(<0.01) | 80.8 |
| MLP | 0.26 | 0.67 | 0.61 | 0.56 | 0.64 | 0.102(<0.01) | 55.2 |
| Average | 0.41 | 0.69 | 0.74 | 0.70 | 0.71 | — | 78.4 |

**Table S4.** Performance of ML models for tick presence estimation on Quebec surveillance sites from 2022-2023, given predictor variables calculated at the surveillance sites using a 1500-meter radius buffer.

| Model | Kappa | Sensitivity | Specificity | F1-score | ROC AUC | Moran's I  (p-value) | % Sites with Non-Clustered Residuals |
| --- | --- | --- | --- | --- | --- | --- | --- |
| XGBoost | 0.47 | 0.84 | 0.64 | 0.74 | 0.74 | -0.0006 (0.20) | 76.1 |
| AdaBoost | 0.46 | 0.87 | 0.55 | 0.73 | 0.72 | 0.045 (<0.01) | 73.5 |
| SVM | 0.46 | 0.92 | 0.55 | 0.74 | 0.75 | 0.065(<0.01) | 73.5 |
| RF | 0.54 | 0.74 | 0.80 | 0.77 | 0.75 | 0.026(0.02) | 80.3 |
| GB | 0.47 | 0.81 | 0.66 | 0.74 | 0.74 | -0.0009(0.19) | 87.2 |
| LR | 0.40 | 0.74 | 0.66 | 0.70 | 0.69 | -0.015(0.28) | 69.2 |
| Ridge Classifier | 0.40 | 0.55 | 0.84 | 0.70 | 0.64 | 0.047 (<0.01) | 73.5 |
| LDA | 0.29 | 0.30 | 0.97 | 0.64 | 0.45 | 0.010(0.07) | 90.6 |
| KNN | 0.25 | 0.49 | 0.76 | 0.62 | 0.55 | 0.069(<0.01) | 77.8 |
| MLP | 0.26 | 0.96 | 0.31 | 0.61 | 0.64 | -0.004(0.28) | 93.2 |
| Average | 0.40 | 0.72 | 0.67 | 0.70 | 0.67 | — | 79.5 |

**Table S5.** Performance of ML models for tick presence estimation on Quebec surveillance sites from 2022-2023, given predictor variables calculated at the surveillance sites using a 2000-meter radius buffer.

| Model | Kappa | Sensitivity | Specificity | F1-score | ROC AUC | Moran's I  (p-value) | % Sites with Non-Clustered Residuals |
| --- | --- | --- | --- | --- | --- | --- | --- |
| XGBoost | 0.44 | 0.65 | 0.80 | 0.68 | 0.61 | -0.002 (0.22) | 67.5 |
| AdaBoost | 0.44 | 0.61 | 0.82 | 0.72 | 0.67 | 0.065 (<0.01) | 80.3 |
| SVM | 0.46 | 0.92 | 0.55 | 0.74 | 0.75 | 0.065(<0.01) | 73.5 |
| RF | 0.49 | 0.75 | 0.74 | 0.75 | 0.73 | 0.022(0.02) | 80.3 |
| GB | 0.37 | 0.62 | 0.73 | 0.65 | 0.64 | -0.010(0.07) | 61.5 |
| LR | 0.35 | 0.68 | 0.67 | 0.68 | 0.66 | -0.017(0.21) | 68.4 |
| Ridge Classifier | 0.42 | 0.92 | 0.51 | 0.72 | 0.74 | 0.059 (<0.01) | 75.2 |
| LDA | 0.26 | 0.27 | 0.97 | 0.62 | 0.41 | 0.002(0.14) | 86.3 |
| KNN | 0.33 | 0.57 | 0.75 | 0.66 | 0.61 | 0.044(<0.01) | 78.6 |
| MLP | 0.52 | 0.88 | 0.65 | 0.76 | 0.77 | -0.016(0.23) | 76.9 |
| Average | 0.41 | 0.68 | 0.72 | 0.70 | 0.65 | — | 74.9 |

**Table S6.** Performance of ML models for tick presence estimation on Quebec surveillance sites from 2022-2023, given predictor variables calculated at the surveillance sites using a 2500-meter radius buffer.

| Model | Kappa | Sensitivity | Specificity | F1-score | ROC AUC | Moran's I  (p-value) | % Sites with Non-Clustered Residuals |
| --- | --- | --- | --- | --- | --- | --- | --- |
| XGBoost | 0.53 | 0.65 | 0.83 | 0.77 | 0.71 | -0.027 (0.02) | 80.0 |
| AdaBoost | 0.46 | 0.88 | 0.58 | 0.74 | 0.72 | 0.111 (<0.01) | 71.8 |
| SVM | 0.44 | 0.89 | 0.58 | 0.73 | 0.73 | 0.071(<0.01) | 70.9 |
| RF | 0.53 | 0.55 | 0.93 | 0.76 | 0.70 | 0.041(<0.01) | 81.8 |
| GB | 0.55 | 0.74 | 0.83 | 0.78 | 0.76 | -0.010(0.06) | 64.5 |
| LR | 0.48 | 0.83 | 0.66 | 0.75 | 0.74 | -0.020(0.111) | 69.1 |
| Ridge Classifier | 0.51 | 0.66 | 0.84 | 0.75 | 0.71 | 0.053 (<0.01) | 82.7 |
| LDA | 0.34 | 0.34 | 0.92 | 0.66 | 0.50 | 0.008(0.07) | 85.5 |
| KNN | 0.28 | 0.50 | 0.78 | 0.64 | 0.57 | 0.034(<0.01) | 75.5 |
| MLP | -0.01 | 0.00 | 0.99 | 0.50 | 0.00 | 0.074(<0.01) | 50.0 |
| Average | 0.41 | 0.60 | 0.79 | 0.70 | 0.61 | — | 73.2 |

**Table S7.** Year-by-year internal cross-validation performance metrics for the models used in this study. Results are based on models trained with predictor variables calculated at the surveillance sites using a 1000-meter radius buffer around each sampling site.

| **Model** | **Year** | **Kappa** | **Sensitivity** | **Specificity** | **ROC AUC** | **F1-score** |
| --- | --- | --- | --- | --- | --- | --- |
| XGBoost | 2014 | 0.59 | 0.71 | 0.88 | 0.80 | 0.82 |
| XGBoost | 2015 | 0.37 | 0.79 | 0.80 | 0.80 | 0.47 |
| XGBoost | 2016 | 0.65 | 0.86 | 0.79 | 0.84 | 0.79 |
| XGBoost | 2017 | 0.48 | 0.78 | 0.75 | 0.78 | 0.65 |
| XGBoost | 2018 | 0.46 | 0.86 | 0.68 | 0.79 | 0.57 |
| XGBoost | 2019 | 0.46 | 0.90 | 0.55 | 0.73 | 0.58 |
| XGBoost | 2020 | 0.57 | 0.95 | 0.57 | 0.77 | 0.64 |
| XGBoost | 2021 | 0.58 | 0.88 | 0.74 | 0.83 | 0.67 |
| AdaBoost | 2014 | 0.03 | 0.98 | 0.05 | 0.51 | 0.09 |
| AdaBoost | 2015 | 0.32 | 0.72 | 0.89 | 0.80 | 0.43 |
| AdaBoost | 2016 | 0.32 | 0.71 | 0.88 | 0.79 | 0.42 |
| AdaBoost | 2017 | 0.33 | 0.58 | 0.87 | 0.72 | 0.56 |
| AdaBoost | 2018 | 0.39 | 0.72 | 0.93 | 0.82 | 0.52 |
| AdaBoost | 2019 | 0.36 | 0.60 | 1.00 | 0.80 | 0.53 |
| AdaBoost | 2020 | 0.47 | 0.89 | 0.61 | 0.75 | 0.56 |
| AdaBoost | 2021 | 0.46 | 0.77 | 0.86 | 0.82 | 0.58 |
| SVM | 2014 | 0.51 | 0.55 | 0.98 | 0.76 | 0.78 |
| SVM | 2015 | 0.27 | 0.68 | 0.89 | 0.78 | 0.40 |
| SVM | 2016 | 0.59 | 0.72 | 0.95 | 0.84 | 0.75 |
| SVM | 2017 | 0.30 | 0.55 | 0.87 | 0.71 | 0.54 |
| SVM | 2018 | 0.31 | 0.64 | 0.93 | 0.78 | 0.46 |
| SVM | 2019 | 0.42 | 0.82 | 0.68 | 0.75 | 0.55 |
| SVM | 2020 | 0.38 | 0.89 | 0.50 | 0.70 | 0.49 |
| SVM | 2021 | 0.45 | 0.76 | 0.86 | 0.81 | 0.58 |
| RF | 2014 | 0.53 | 0.86 | 0.66 | 0.76 | 0.73 |
| RF | 2015 | 0.58 | 0.92 | 0.78 | 0.85 | 0.64 |
| RF | 2016 | 0.54 | 0.95 | 0.55 | 0.75 | 0.66 |
| RF | 2017 | 0.48 | 0.97 | 0.43 | 0.70 | 0.57 |
| RF | 2018 | 0.48 | 0.93 | 0.53 | 0.73 | 0.55 |
| RF | 2019 | 0.41 | 0.95 | 0.39 | 0.67 | 0.49 |
| RF | 2020 | 0.23 | 0.99 | 0.17 | 0.58 | 0.27 |
| RF | 2021 | 0.43 | 0.89 | 0.55 | 0.72 | 0.53 |
| GB | 2014 | 0.54 | 0.73 | 0.82 | 0.77 | 0.77 |
| GB | 2015 | 0.39 | 0.82 | 0.78 | 0.80 | 0.48 |
| GB | 2016 | 0.58 | 0.87 | 0.70 | 0.79 | 0.71 |
| GB | 2017 | 0.51 | 0.84 | 0.70 | 0.77 | 0.64 |
| GB | 2018 | 0.44 | 0.86 | 0.67 | 0.77 | 0.54 |
| GB | 2019 | 0.41 | 0.92 | 0.46 | 0.69 | 0.51 |
| GB | 2020 | 0.59 | 0.97 | 0.56 | 0.76 | 0.65 |
| GB | 2021 | 0.59 | 0.90 | 0.73 | 0.82 | 0.67 |
| LR | 2014 | 0.54 | 0.71 | 0.84 | 0.77 | 0.77 |
| LR | 2015 | 0.26 | 0.66 | 0.89 | 0.78 | 0.39 |
| LR | 2016 | 0.48 | 0.78 | 0.73 | 0.75 | 0.66 |
| LR | 2017 | 0.22 | 0.62 | 0.65 | 0.64 | 0.47 |
| LR | 2018 | 0.31 | 0.70 | 0.80 | 0.75 | 0.45 |
| LR | 2019 | 0.25 | 0.70 | 0.64 | 0.67 | 0.43 |
| LR | 2020 | 0.29 | 0.71 | 0.72 | 0.72 | 0.45 |
| LR | 2021 | 0.27 | 0.62 | 0.82 | 0.72 | 0.46 |
| Ridge Classifier | 2014 | 0.56 | 0.69 | 0.89 | 0.79 | 0.79 |
| Ridge Classifier | 2015 | 0.33 | 0.73 | 0.89 | 0.81 | 0.44 |
| Ridge Classifier | 2016 | 0.68 | 0.89 | 0.80 | 0.84 | 0.79 |
| Ridge Classifier | 2017 | 0.44 | 0.77 | 0.74 | 0.75 | 0.61 |
| Ridge Classifier | 2018 | 0.31 | 0.70 | 0.80 | 0.75 | 0.45 |
| Ridge Classifier | 2019 | 0.42 | 0.72 | 0.89 | 0.80 | 0.57 |
| Ridge Classifier | 2020 | 0.37 | 0.70 | 0.89 | 0.79 | 0.52 |
| Ridge Classifier | 2021 | 0.36 | 0.78 | 0.68 | 0.73 | 0.50 |
| LDA | 2014 | 0.66 | 0.84 | 0.82 | 0.83 | 0.82 |
| LDA | 2015 | 0.45 | 0.82 | 0.89 | 0.85 | 0.53 |
| LDA | 2016 | 0.49 | 0.96 | 0.48 | 0.72 | 0.61 |
| LDA | 2017 | 0.25 | 0.97 | 0.22 | 0.59 | 0.33 |
| LDA | 2018 | 0.12 | 0.95 | 0.13 | 0.54 | 0.19 |
| LDA | 2019 | 0.02 | 0.98 | 0.04 | 0.51 | 0.06 |
| LDA | 2020 | 0.55 | 0.89 | 0.72 | 0.81 | 0.63 |
| LDA | 2021 | 0.39 | 0.98 | 0.32 | 0.65 | 0.45 |
| KNN | 2014 | 0.44 | 0.84 | 0.59 | 0.72 | 0.67 |
| KNN | 2015 | 0.32 | 0.90 | 0.44 | 0.67 | 0.40 |
| KNN | 2016 | 0.59 | 0.88 | 0.70 | 0.79 | 0.72 |
| KNN | 2017 | 0.53 | 0.81 | 0.78 | 0.80 | 0.67 |
| KNN | 2018 | 0.44 | 0.89 | 0.60 | 0.74 | 0.53 |
| KNN | 2019 | 0.14 | 0.96 | 0.14 | 0.55 | 0.22 |
| KNN | 2020 | 0.40 | 0.98 | 0.33 | 0.66 | 0.46 |
| KNN | 2021 | 0.55 | 0.95 | 0.55 | 0.75 | 0.62 |
| MLP | 2014 | 0.00 | 1.00 | 0.00 | 0.50 | 0.00 |
| MLP | 2015 | 0.10 | 0.32 | 1.00 | 0.66 | 0.27 |
| MLP | 2016 | 0.47 | 0.80 | 0.68 | 0.74 | 0.65 |
| MLP | 2017 | 0.00 | 1.00 | 0.00 | 0.50 | 0.00 |
| MLP | 2018 | 0.00 | 1.00 | 0.00 | 0.50 | 0.00 |
| MLP | 2019 | 0.46 | 0.68 | 0.78 | 0.73 | 0.66 |
| MLP | 2020 | 0.43 | 0.82 | 0.72 | 0.77 | 0.54 |
| MLP | 2021 | 0.21 | 0.47 | 0.95 | 0.71 | 0.43 |

**Table S8.** Summary of average model performance (± standard deviation) across years 2014–2021. Results are based on models trained with predictor variables calculated at the surveillance sites using a 1000-meter radius buffer around each sampling site.

| Model | Kappa | Sensitivity | Specificity | F1-score | ROC AUC |
| --- | --- | --- | --- | --- | --- |
| XGB | 0.52 (0.092) | 0.84 (0.077) | 0.72 (0.113) | 0.65 (0.144) | 0.79 (0.033) |
| AdaBoost | 0.34 (0.136) | 0.75 (0.135) | 0.76 (0.308) | 0.46 (0.161) | 0.75 (0.103) |
| SVM | 0.40 (0.111) | 0.70 (0.123) | 0.83 (0.163) | 0.57 (0.135) | 0.77 (0.048) |
| RF | 0.46 (0.11) | 0.93 (0.041) | 0.51 (0.183) | 0.56 (0.137) | 0.72 (0.077) |
| GB | 0.51 (0.082) | 0.86 (0.073) | 0.68 (0.116) | 0.62 (0.101) | 0.77 (0.037) |
| LR | 0.33 (0.117) | 0.69 (0.051) | 0.76 (0.089) | 0.51 (0.132) | 0.73 (0.049) |
| Ridge Classifier | 0.44 (0.129) | 0.75 (0.067) | 0.82 (0.081) | 0.58 (0.137) | 0.78 (0.037) |
| LDA | 0.37 (0.221) | 0.92 (0.065) | 0.45 (0.327) | 0.45 (0.249) | 0.69 (0.135) |
| KNN | 0.43 (0.146) | 0.90 (0.058) | 0.52 (0.206) | 0.54 (0.169) | 0.71 (0.082) |
| MLP | 0.15 (0.197) | 0.80 (0.267) | 0.42 (0.461) | 0.24 (0.273) | 0.61 (0.122) |
